# A Kinetic Study of Accumulation and Elimination of Microcystin-LR in Yellow Perch (*Perca Flavescens*) Tissue and Implications for Human Fish Consumption

**DOI:** 10.3390/md9122553

**Published:** 2011-12-08

**Authors:** Julianne Dyble, Duane Gossiaux, Peter Landrum, Donna R. Kashian, Steven Pothoven

**Affiliations:** 1 NOAA Great Lakes Environmental Research Laboratory, 4840 South State Road, Ann Arbor, MI 48108, USA; Email: duane.gossiaux@noaa.gov (D.G.); plandrumprof@charter.net (P.L.); 2 Department of Biological Sciences, Wayne State University, 5047 Gullen Mall, Detroit, MI 48202, USA; 3 Lake Michigan Field Station, NOAA Great Lakes Environmental Research Laboratory, 1431 Beach Street, Muskegon, MI 49441, USA; Email: steve.pothoven@noaa.gov

**Keywords:** microcystin, accumulation, kinetics, yellow perch, *Microcystis*, Great Lakes, fish consumption

## Abstract

Fish consumption is a potential route of human exposure to the hepatotoxic microcystins, especially in lakes and reservoirs that routinely experience significant toxic *Microcystis* blooms. Understanding the rates of uptake and elimination for microcystins as well as the transfer efficiency into tissues of consumers are important for determining the potential for microcystins to be transferred up the food web and for predicting potential human health impacts. The main objective of this work was to conduct laboratory experiments to investigate the kinetics of toxin accumulation in fish tissue. An oral route of exposure was employed in this study, in which juvenile yellow perch (*Perca flavescens*) were given a single oral dose of 5 or 20 μg of microcystin-LR (MC-LR) via food and accumulation in the muscle, liver, and tank water were measured over 24 h. Peak concentrations of the water soluble fraction of microcystin were generally observed 8–10 h after dosing in the liver and after 12–16 h in the muscle, with a rapid decline in both tissues by 24 h. Up to 99% of the total recoverable (*i.e.*, unbound) microcystin was measured in the tank water by 16 h after exposure. The relatively rapid uptake and elimination of the unbound fraction of microcystin in the liver and muscle of juvenile yellow perch within 24 h of exposure indicates that fish consumption may not be a major route of human exposure to microcystin, particularly in the Great Lakes.

## 1. Introduction

Cyanobacterial harmful algal blooms (HABs) are increasingly prevalent in the Great Lakes as well as smaller inland lakes and reservoirs throughout the world, fueled by eutrophication and perhaps increasing global temperatures [1,2]. The proliferation of HABs has significant ecological consequences, including contributing to hypoxia and food web disruption [3]. In addition, there are also significant concerns about the direct impacts of cyanotoxins on human and animal health. In shallow, warm, nutrient enriched regions of the North American Great Lakes, such as the western basin of Lake Erie, Saginaw Bay (Lake Huron), and Green Bay (Lake Michigan), summer HAB blooms dominated by *Microcystis* are common and produce the hepatotoxic microcystins [4,5]. Microcystins are a group of cyclic peptide toxins whose variants differ in the L-amino acid residues 2 (X) and 4 (Z) [6]. The most commonly occurring variant, as well as one of the most toxic, is microcystin-LR (MC-LR) and the World Health Organization has established a provisional guideline for MC-LR in finished drinking water of 1 µg L^−1^ [7]. Microcystins inhibit serine/theonine protein phosphatases [6] which can cause disintegration of the liver structure, cellular damage to hepatocytes, liver necrosis, and internal hemorrhage in the liver [8]. Symptoms of acute microcystin exposure in humans has included gastroenteritis (vomiting, diarrhea, abdominal cramping) and blistering around the mouth and pneumonia during incidences of immersion [9,10,11]. 

Less studied are the potential consequences of low-level chronic exposure, which could result in liver disease, promotion of carcinogenic tumors in animal models [12] and is perhaps linked to liver and colon cancer [13,14]. Chronic exposure of humans to microcystins has been considered primarily through drinking water, which has been correlated with increased liver damage in local residents [9,13] and recreational contact in waters experiencing *Microcystis* blooms [15]. Another potential route of human exposure that has not been widely investigated is fish consumption. Recreational and commercial fishing is particularly important in the Great Lakes, including in areas with frequent and dense *Microcystis* blooms. Fish can be a significant part of the local diet and it is not well known the degree to which microcystins accumulate in edible muscle tissue of popular Great Lakes recreational fish (e.g., yellow perch, walleye, and bluegill). Microcystins are highly stable compounds and toxicity is not reduced by boiling or cooking [16]. More data on the timing and concentrations at which microcystins accumulate in fish muscle will provide a better understanding of the potential for fish to be a route of chronic exposure of humans to cyanotoxins. 

It is generally agreed that the major route in which microcystins are taken up by fish is through the gastrointestinal tract and therefore primarily via diet [17]. There is some debate in the literature about the potential for microcystins to pass through fish gills. Some studies observed bioaccumulation of microcystins on gills and damage to gill tissue by MC-LR [18,19], while others maintain the microcystin compounds are too large to cross the gill membrane [20,21]. Phytoplanktivorous fish may take up toxic *Microcystis* cells directly from the water column [22], but likely many fish are exposed through their diet of zooplankton, benthic invertebrates, and fish [19,23]. The degree to which *Microcystis* cells pass through the fish gut and are excreted unabsorbed is also not well known. Carbis *et al.* [24] demonstrate mucus production in the intestine upon exposure to microcystins, which might block hepatatoxin uptake. However, it is clear that uptake of microcystins is not always completely blocked since fresh- and brackish-water fishes are known to accumulate cyanotoxins in tissues, including muscle, viscera, heart, intestine, gallbladder, spleen, gonad, brain and liver [23,25,26,27,28,29]. Most of the studies that assess uptake of microcystins in fish used intraperitoneal (IP) injection as the route of exposure. While useful in delivering a known dose of toxin, IP injection does not provide an environmentally realistic route of exposure to the fish and represents direct delivery of the toxin instead of the usual route through the intestines. Thus, the IP route may not provide an accurate assessment of the rates and locations of toxin accumulation in the fish. Other studies have measured microcystin concentrations in wild-caught fish inhabiting water bodies with cyanoblooms, including western Lake Erie [28,30]. An inherent difficulty in trying to correlate concentrations in fish tissue to lake concentrations of microcystins is the heterogeneity in exposure due the capacity of fish to swim in and out of *Microcystis* blooms, making the dose of microcystins received impossible to ascertain. Thus, while measurements of concentrations of microcystins in field-collected fish are useful in identifying whether this is a potential route for human exposure, it reveals less about the mechanism of accumulation and how rapidly the fish will eliminate the absorbed microcystins. This study used a single known oral dose of the most commonly occurring microcystin variant, MC-LR, in yellow perch to investigate the kinetics of toxin uptake and elimination in fish muscle and liver tissue over a short (24 h) time course. The goal of this study is to better understand whether fish consumption is a potential route of human exposure to microcystins.

## 2. Results and Discussion

### 2.1. Timing of MC-LR Accumulation in Yellow Perch

The preliminary experiment that was used to select the appropriate time points for sampling fish after MC-LR exposure showed that after a dose of 5 µg·fish^−1^, MC concentration in both the liver and muscle peaked at 8–10 h and returned to concentrations below 4 ng g^−1^ MC within 24 h, remaining low through the end of the experiment at 240 h (unpublished data). This preliminary experiment suggested that a more detailed analysis of time points up through 24 h should be the focus of the dosing experiments, which resulted in an increased number of time points sampled between 0–24 h and no further sampling past 24 h in the remainder to the experiments, described below. 

Environmentally relevant doses were chosen for the experiments. Yellow perch less than 150 mm in size generally consume 1.31 g dry food per 100 g fish (wet weight) each day [31] and it is known from stomach content analysis by Wilson *et al.* [28] that early in the summer, juvenile yellow perch in western Lake Erie are eating predominantly benthic invertebrates (76%), followed by fish (18%) and zooplankton (7%). The total weight of food consumed was scaled to the average fish size in this experiment and partitioned based on this expected diet. Literature sources were used to identify some minimum and maximum concentrations of microcystins commonly found in those groups via natural routes of exposure. Concentrations of microcystins ranged 1–30 µg g^−1^ in benthic macroinvertebrates in Michigan lakes with *Microcystis*, 0.017–1.19 µg g^−1^ in the muscle and liver of Lake Erie juvenile yellow perch and 0.2–1352 µg g^−1^ in zooplankton (mostly *Daphnia*) [17,28]. Although these are approximations and the exact concentration of microcystins in each component of the diet is not known, particularly for chironomids which are a significant part of the diet at this life stage, daily exposure to microcystins for this size fish via an early summer diet during a toxic *Microcystis* bloom is estimated to be in the range of 1–25 µg for yellow perch. 

The dose of MC-LR given on each food pellet was confirmed by extraction and quantification by ELISA from spare pellets. The 5 µg MC-LR dose was actually 6.38 ± 0.58 µg and the 20 µg dose was 19.70 ± 2.20 µg. The analytical error was determined by quantifying the recovery of a known concentration of MC-LR spiked into control tissue samples before extraction. The recovery of spiked MC-LR into muscle tissue was 89.3 ± 9.3% (*n* = 11) and from liver tissue was 77.0 ± 10.6% (*n* = 4). The liver and muscle MC data presented below have not been corrected for these recovery rates. 

Yellow perch of similar size were chosen for the experiments, but there was still variability in fish weight within and between experiments. Fish in the 5 µg experiment were 12.61 ± 4.80 g wet weight (range of 5 to 24.5 g) and in the 20 µg experiment were 17.62 ± 5.65 g wet weight (range of 9.6 to 29.3 g) even though the lengths only varied by a factor of 2.1 for the 5 µg dose and 1.4 for the 20 µg dose. Thus, the condition of the fish may have been different even though they were from the same source. Fish of smaller size are known to have a higher metabolism and potentially higher concentrations of contaminant relative to similarly exposed fish of a larger size [32,33]; therefore the lack of uniform fish size may have increased the variability of MC concentrations between replicate fish. To control for this variability and better assess the impacts of time on MC accumulation in fish tissues, the lowest 5% of all fish in each experiment by weight were not included in the data analysis to determine peak periods of MC concentrations in the tissues. This was a total of three fish per experiment. In addition, one fish per experiment was rejected as an outlier based on both tissue and liver concentrations (*Q*-test, 95% confidence interval) [34]. 

**Figure 1:**
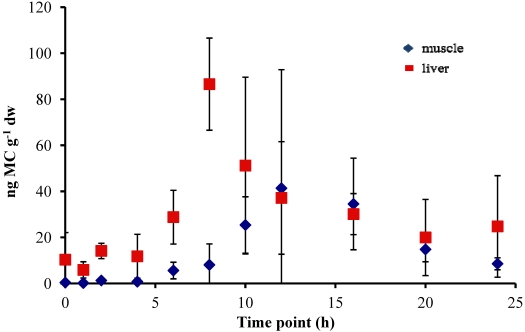
Concentrations of microcystin (ng microcystin per g dw fish tissue) in yellow perch liver and muscle tissue for time points 0–24 h after given a single oral dose of 5 µg MC-LR. Error is expressed as standard deviation of four replicate fish.

**Figure 2:**
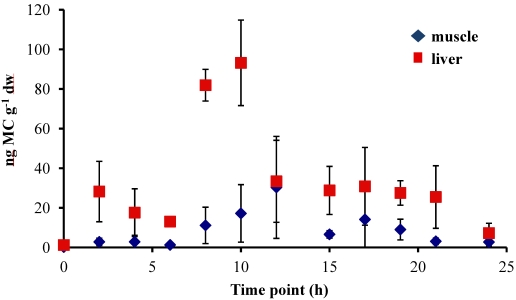
Concentrations of microcystin (ng microcystin per g dw fish tissue) in yellow perch liver and muscle tissue for time points 0–24 h after given a single oral dose of 20 µg MC-LR. Error is expressed as standard deviation of four replicate fish.

In all experiments, the concentration of MC in the fish liver was elevated by 4–6 h and peaked at 8–10 h after oral dosing. In the 5 µg experiment, there was a sharp increase at 8 h to 86.58 ± 20.01 ng MC g^−1^ dw (dry weight), followed by a significant decrease to less than 38 ng MC g^−1^ dw by 12 h (Figure 1). In the 20 µg experiment, the MC concentration in the liver remained elevated at 8–10 h, with a peak concentration of 93.19 ± 21.56 ng MC g^−1^ dw, and then decreased to less than 35 ng MC g^−1^ dw by 12 h post-dosing (Figure 2). In both the 5 µg and 20 µg dose experiments, the concentration in the liver remained between 20–30 ng MC g^−1^ dw through 21 h post-dosing. By 24 h, the liver MC concentration remained at 24.78 ± 22.04 ng MC g^−1^ dw in the 5 µg experiment, but in the 20 µg dose decreased further to 7.27 ± 4.96 ng MC g^−1^ dw. At 24 h, MC concentrations in the liver of control fish *not* dosed with MC was 9.03 ± 4.14 ng MC g^−1^ dw for the 5 µg experiment and 0.72 ± 0.54 ng MC g^−1^ dw for the 20 µg experiment. 

Concentrations of MC in the fish muscle tissue peaked at 12 h after the fish was dosed in both the 5 µg and 20 µg experiment. Concentrations were lower in the muscle than in the liver, with maximum concentrations of 41.38 ± 51.49 ng MC g^−1^ dw in the 5 µg experiment (Figure 2) and 30.35 ± 25.75 ng MC g^−1^ dw in the 20 µg experiment (Figure 2). By 24 h post-dosing, the average MC concentrations in the 5 µg experiment (Figure 1) was 8.54 ± 2.58 ng MC g^−1^ dw and 2.74 ± 2.14 ng MC g^−1^ dw for the 20 µg experiment (Figure 2). The muscle tissue of control fish at the 24 h time point contained 0.08 ± 0.06 ng MC g^−1^ dw for the 5 µg experiment (Figure 1) and 1.81 ± 1.03 ng MC g^−1^ dw in the 20 µg experiment (Figure 2).

Initial concentrations of MC were also higher in fish from the 5 µg experiment in comparison to the 20 µg experiment. At the beginning of the experiment, before oral dosing, MC concentrations in the liver and muscle were 10.36 ± 11.75 and 0.38 ± 0.22 ng MC g^−1^ dw, respectively, in the 5 µg experiment and 1.25 ± 0.76 and 0.21 ± 0.10 ng MC g^−1^ dw in the 20 µg experiment. As a hepatotoxin targeting the liver, at least some portion of any measurable accumulation of MC in the fish organs would be expected in the liver tissue. In this study, an initial peak in MC concentrations was measured in the liver at 8–10 h followed by maximum MC accumulation in the muscle tissue at 12 h and a significant decrease in the concentration of unbound MC in both tissues by 24 h after exposure. The measurement of maximum MC concentrations in the liver that were as much as three times higher than those in the muscle also further supports the preferential accumulation of this toxin in the liver. Multiple field studies have documented the uptake of microcystins into the liver and muscle of planktivorous [22,23], omnivorous [18,19] and piscivorous fish [19] and lab studies using fish dosed through oral gavage or intraperitoneal (IP) injection [29,35,36,37] have confirmed the accumulation of microcystins in these organs. However, despite the useful data these studies have contributed, there still remains an incomplete understanding of the capacity of fish to accumulate and eliminate microcystins and the timing over which this occurs. This is in part due to the difficulty in determining the actual levels of exposure in field-caught fish and the lack of environmentally relevant modes of exposure in laboratory studies. While it is acknowledged that the current study also lacks some environmental relevance due to the use of purified toxin instead of microcystins delivered in the form of *Microcystis* cells, or other food source which has accumulated toxins by feeding or filtering, the advantage here is that a known dose of MC-LR is provided, eliminating variability associated with cell size, mucilage content, internal microcystin content or other matrix effects of *Microcystis* cells. 

The current study assessed the effect of a single dose, in contrast to many of the previous feeding studies in which fish were exposed to MC-LR over a multiple day period [38,39]. A single dose was chosen in order to better determine the timing of uptake into tissues. However, despite the differences in feeding frequency and experiment duration between this and other studies, the pattern of accumulation of microcystins in the liver first followed by the muscle is relatively consistent. In a 15 day study in which juvenile tilapia were fed *Microcystis* cells daily containing concentrations of microcystins in a similar range to our 20 µg MC-LR dose, maximum liver concentrations of microcystins were observed on day 6 and maximum muscle concentrations on day 9 [38]. In a similar experiment, Smith and Haney [39] measured the accumulation and elimination of microcystins in pumpkinseed sunfish using a 1000 fold lower dose of microcystins given over 9 days via a zooplankton food source. Their first trial showed a similar pattern of accumulation with the maximum concentration of microcystins found in the liver at 2–4 days and in the muscle by day 4. However, the liver and muscle concentrations of microcystins did not show this clear pattern in their second trial, likely due to the high degree of variability measured in samples [39], which also highlights the heterogeneity in response between individual fish. Such heterogeneity among fish may be due to size, as observed in this study.

Two different doses of MC-LR were administered in this study, but there was not a clear dose dependent accumulation detected. Maximum MC concentrations in both the liver and muscle tissues were similar in the 5 µg and 20 µg doses, as was the timing of accumulation and elimination of the toxin. While this could be due to the maximum capacity of the tissues to absorb the compound, there were also a number of other confounding factors that may have impacted the measured MC concentrations. The presence of microcystins in the fish livers of the fish at the 0 h time point (before dosing) in the 5 µg experiment was evidence that the fish had previous exposure to microcystins in the fish farm from which they originated. Although the fish were held in microcystin-free water for 24–28 h before the start of the experiment, low levels of microcystins in the liver persisted, likely resulting in higher maximum MC concentrations than would have otherwise been measured. Additionally, the fish used in the 5 µg experiment were smaller on average than those used in the 20 µg experiment. A previous study [40] also observed that smaller fish have higher concentrations of microcystins than larger fish of the same species from the same lake. This difference in mass between dosing groups is potentially a main driving force in tissue concentrations of microcystins. A fish with more mass will require a greater accumulation of toxin to achieve a higher tissue concentration. 

As pointed out by Smith and Haney [39], the amount of microcystins that accumulate in fish may be determined by the route by which the fish is exposed. By 24 h of exposure, 80% of microcystins administered to pumpkinseed sunfish through a zooplankton diet was accumulated in a non-covalently bound form in the liver [39], while there was only approximately 1.7–10% absorption in the liver of MC-LR delivered to rainbow trout via gavage of toxic *Microcystis* cells [20,35], 5% absorption of MC-LR in the livers of yellow perch via an oral dose of MC-LR (this study) and 0.3% uptake in the liver when rainbow trout were orally gavaged with purified MC [36]. While the purified MC-LR would be expected to be more available for uptake than intracellular microcystin, it is also possible that much of the extracellular microcystin passes directly through the gut and is excreted without being taken up into the organ tissues. The peptide bonds linking D-amino acids in microcystins are not susceptible to normal hydrolytic enzymes, making these toxins resistant to digestion in the gastrointestinal tract [41,42]. The microcystin congener also impacts the degree to which microcystins are taken up by the fish. MC-LR alone was used as the microcystin dose in this study and there is an indication that MC-LR may be taken up to a much lower degree than microcystin-RR, another of the 80+ variants of microcystin. Xie *et al.* [22] measured microcystins in silver carp (*Hypophthalmichthys molitrix*) fed toxic *Microcystis viridis* cells containing both MC-LR and -RR and, despite a high concentration of MC-RR in blood, liver, muscle and intestines, MC-LR was only found in the intestines of these fish. Comparing the ratio of MC-LR:RR in the seston *vs.* fish gut and feces suggested that the transport of MC-LR across the intestines is selectively inhibited, but MC-RR is able to cross through the intestines and into muscle tissues [22,43]. MC-RR was also measured in trace amounts in the brain of fish (*Jenynsia multidentata*) exposed to dissolved MC-RR in the laboratory, in addition to accumulating in the muscle at the end of the 24 h exposure [18]. *Microcystis* blooms generally contain multiple microcystin congeners so determining which are present may be important to assess potential toxicity and accumulation in fish. Therefore, despite the lower toxicity of MC-RR compared to many other microcystin variants, including MC-LR [44], its increased bioavailability may result in increased transport through trophic levels [22]. Though there is no evidence for biomagnification of microcystins in the food web, this toxin is vectorially transported to higher trophic levels [23]. Thus, the trophic level at which fish feed is also an important indicator of their potential tissue toxin concentration. There is no consensus in the literature about whether phytoplanktivorous or piscivorous fish generally have the highest levels of microcystins [19,23,45] and, even within these groups, there are likely significant species specific differences in metabolism of microcystins [30,46]. 

### 2.2. Microcystin Elimination from Yellow Perch and Mass Balance

**Figure 3:**
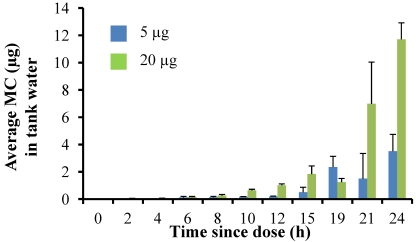
Average microcystin concentration (µg MC) in fish tanks for time points 0–24 h after fish given a single oral dose of MC-LR (either 5 µg or 20 µg dose). This concentration represents the MC excreted by the fish, including both dissolved MC and any feces present. Error is expressed as standard deviation of four replicate fish tanks.

Dissolved MC concentrations in the tank water increased with time. Microcystins were measurable in the tank water by 4 h after the fish were dosed in both the experiments and then steadily increased over the course of the experiment, with the highest concentrations measured at 24 h. In the 5 µg dose experiment, the average MC concentration in the tank water was 0.50 ± 0.37 µg at 12 h and 3.5 ± 1.2 µg by 24 h (Figure 3). Overall, concentrations were higher in the 20 µg dose experiment, with an average dissolved MC concentration in the tank water of 1.01 ± 0.11 µg at 12 h and 11.71 ± 1.2 µg by 24 h (Figure 3). 

In order to evaluate how the MC is partitioned, a rough mass balance was calculated by summing the total amount of MC in the fish muscle, liver, and tank water for each time point. Total MC measured in the fish and tank water is almost 3% of the initial 5 µg dose at 4 h, 10.5% by 12 h and a maximum of 70.3% accounted for by 24 h. In the 20 µg experiment, the total MC in fish liver, muscle and tank water combined was less than 1% of the initial dose at 4 h, less than 10% through 16 h and was 58.6% of the initial dose by 24 h (Table 1). Microcystins in the liver and muscle tissues never exceeded 0.3% of the total initial dose. 

**Table 1:** Percent of total microcystin dose measured at selected time points.

Time point (h)	5 µg			20 µg		
	Muscle	Liver	Tank	Muscle	Liver	Tank
**4**	0.01	0.04	2.65	0.01	0.01	0.27
**8**	0.03	0.05	3.41	0.03	0.03	1.27
**10**	0.16	0.04	3.76	0.04	0.05	3.21
**12**	0.29	0.09	10.10	0.10	0.08	5.04
**16**	0.22	0.03	47.02	0.02	0.01	9.19
**24**	0.07	0.02	70.17	0.01	0.01	58.56

Microcystin concentrations in the digestive track were not measured in this study, so it is not known what percentage of the total MC-LR dose passed through the gut unabsorbed compared to the percentage taken up by the tissues and subsequently detoxified and excreted within 24 h. To find out how quickly unabsorbed MC-LR might be expected to pass through the fish gut, an evacuation rate model was used. This model predicts the digestion of food pellets under given temperatures and fish sizes [31]. For the conditions of this experiment (20 °C, 15 g fish, 1 g food pellet), this model predicts that by 24 h at least 90% of the food pellet would have been excreted, thus suggesting that at least 90% of the MC-LR not taken up into fish tissue would have been passed through the gut without being absorbed by the end of the experiment. This rapid passage of food through the gut based on the digestion model suggests a relatively low accumulation efficiency for MC from food into the fish tissues. The peak concentrations observed with a single exposure were somewhat lower than the average liver concentration for field collected perch but much greater for muscle tissue than found in the field [28]. However, the rapid elimination rate from the tissue is independent of the uptake once exposure has ceased (e.g., the end of the ingestion of contaminated prey). 

Even given this rapid passage of food, and potentially associated MC, through the gut, there was still at least 30% of the initial MC-LR dose that was unaccounted for in the muscle, liver and tank water by the end of this experiment. A small percentage of this dose may be found in bile or other organs, including the heart, gonads, and stomach, but the literature suggests this is minimal [17,47,48]. A more significant percentage of the unaccounted fraction of the MC-LR dose is likely bound covalently and irreversibly to protein phosphatase 1 and 2A. The solvent extraction used in this study will not reverse the covalent bonds that can be formed between the MC-LR and the protein phosphatase enzyme active site and thus is only a measure of the unbound MC fraction, which may underestimate the true toxin load to the tissues (John Berry, Florida International University, pers. comm.). Previous studies have shown that as little as 24% of the total microcystins could be extracted from Atlantic salmon liver [49]. 

The degree to which microcystins covalently bound to fish tissue is biologically available to higher trophic levels, including human consumers, is unknown [23,42]. However, it is likely that the unbound fraction is more readily available for trophic transfer. Of the total *recoverable* (*i.e.*, unbound fraction) of MC measured in fish tissues and tank water in this experiment, the amount distributed in the muscle and liver tissues is not more than 4.1% at any given time point. A very high percentage of the recovered MC was in the tank water fraction, which also included any feces that might have been present. For both doses, from 15 h through the end of the experiment, the MC in the tank water comprised >99% of the total unbound MC. This large percentage of the unbound MC found in the tank water by 24 h after exposure to a single oral dose indicates that human exposure to microcystin via consumption of yellow perch is reduced as long as just the muscle tissue is being consumed. Since in many cases fish are only exposed to cyanotoxins intermittently while swimming through patchy blooms, there may be sufficient opportunity for elimination before being caught for human consumption. In addition to the decline in measurable microcystins in the liver and muscle by 24 h after exposure, the hepatic effects on fish are short term and serum biochemistry and hepatocyte morphology return to normal within 30 days of removal from exposure [24,37]. However, in small lakes with persistent toxic blooms, regular exposure may increase the probability of microcystins being present in the tissues of resident fish at the time they are caught for consumption. In a study on wild caught yellow perch in western Lake Erie, which experiences regular summertime blooms of *Microcystis*, maximum concentrations of microcystins of 4.02 ng g dw^−1^ in the muscle tissue were measured over the course of the bloom season [28], which is 8.6 times lower than the maximum muscle MC concentrations measured in this study. The maximum concentrations of microcystins measured in the liver of these wild caught fish was 12.7 times higher than in this experiment (1182 ng·g·dw^−1^), suggesting that the lower toxin load in the muscle tissue was not solely due to a lower absorption of microcystins. There are many other commercially and recreationally important fish in the Great Lakes and a recent study has measured higher concentrations of microcystins in the muscle of wild caught fish belonging to these groups, particularly walleye, white bass, and smallmouth bass from Lake Erie (maximum concentrations of 43.6 ng·g·wet weight^−1^) and alewives and northern pike from the Bay of Quinte, Lake Ontario (maximum concentrations of 37.5 ng·g·wet weight^−1^) [40]. Naturally, the amount of microcystins that fish will be exposed to in their natural environment will vary greatly with bloom dynamics and toxicity, frequency and duration of exposure, fish size and diet, timing and frequency of feeding, food availability, life history, *etc.*

### 2.3. Kinetics of MC Uptake

The data were difficult to kinetically model in the sense that they were extremely variable (Figure 1 and Figure 2). The variability could have been due to several sources of uncertainty. First, an examination of analytical variation yielded a relative percent difference for muscle tissue, measured in duplicate, of 41.1 ± 36.6% for the 5 µg dose and 60.5 ± 37.7% for the 20 µg dose. While this analytical variation appears to be large, it was not sufficient to account for most of the variation in the observed data, which were up to ten-fold or more different among tissue concentrations of different fish sampled after the same length of exposure (Figure 1 and Figure 2). This variation appears to in part be due to differences in fish size, with smaller fish exhibiting greater tissue concentrations after the same length of exposure. 

As a result of the above variations in concentration, the fit to the data for the two tissues were not as robust as preferred but do provide some insight into the rate processes for microcystin in these fish. First, the elimination is relatively rapid with an apparent elimination half-life (elimination *t*_0.5_) calculated from *k_e_* in the range of 3.3 to 7.8 h for muscle (Table 2). Given this, the fish would be expected to eliminate 95% of the accumulated MC from muscle in 14.5 to 33.5 h (elimination *t*_0.05_). The elimination half-life for MC in the liver was calculated to be 10.5 h, with 95% elimination from the fish occurring within 46 h. The elimination modeled in this study does not specify mechanism but includes actual loss from the tissue as well as biotransformation to an unextractable or unmeasurable form.

The loss rates for the exposure dose are the rates at which exposure of the fish to the initial dose decreases as the MC is taken up into the fish tissue or eliminated without being absorbed and no longer can serve as a source of exposure. These apparent loss rates for the exposure dose are similar between dose levels but different between tissues. The apparent loss of exposure was modeled as a faster (larger dose loss rate, λ) for the liver than for the muscle, meaning the period of time in which the liver was exposed to MC was shorter than the period of time the muscle was exposed. Microcystin-LR exposure in the liver has a shorter half-life (exposure *t*_0.5_) (2.3–4.5 h) compared to the muscle (14.8–16 h) due to the lower loss rate for exposure in the muscle. This suggests that 95% of the exposure to the initial dose (exposure *t*_0.05_) is complete by 9.9–19.3 h in the liver but not until 63.7–69.2 h in the muscle (Table 2). This supports our approach of determining the elimination rate constant as a simple first order model since uptake would have been low and likely insignificant by 10 to 12 h, considering the very small size of the uptake rate constants. 

**Table 2:** Kinetic parameter estimates for MC in yellow perch for each tissue at each exposure dose. *k_e_* is the elimination rate constant (h^−1^), *k_u_* is the uptake rate constant (g^−1^·h^−1^), λ is the loss rate for the dose (h^−1^), elimination t_0.5_ is the half-life in each tissue, elimination *t*_0.05_ is the time for 95% elimination from the tissue (both elimination values calculated based on *k_e_*), exposure *t*_0.5_ is the half-life for exposure in each tissue, exposure *t*_0.05_ is the time for completion of 95% of the exposure (both exposure values calculated based on λ).

Tissue/Concentration	*k_e_* (h^−1^)	*k_e_* r^2^	*k_u_* (g^−1^h^−1^)	λ (h^−1^)	*k_u_*, λ r^2^	Elimination		Exposure	
						*t*_0.5_ (h)	*t*_0.05_ (h)	*t*_0.5_ (h)	*t*_0.05_ (h)
5 µg–Muscle	0.089 ± 0.058	0.55	0.00058 ± 0.00024	0.047 ± 0.051	0.41	7.8	33.5	14.8	63.7
20 µg–Muscle	0.21 ± 0.07	0.62	0.00017 ± 0.000059	0.043 ± 0.035	0.44	3.3	14.5	16.0	69.2
5 µg–Liver	0.066 ± 0.037	0.71	0.0019 ± 0.0006	0.155 ± 0.069	0.59	10.5	45.4	4.5	19.3
20 µg–Liver	0.066 ± 0.029	0.81	0.0016 ± 0.0007	0.30 ± 0.15	0.55	10.5	45.8	2.3	9.9

The uptake rate constants (*k_u_*) calculated in this model are somewhat different from more familiar values that are often calculated, such as clearance coefficients. The uptake rate constants indicate the fraction of the dose accumulated by the respective fish tissue each hour. As with the exposure constants, the uptake rate constants are similar between doses but different between tissues. The uptake of the liver is relatively fast, about ten times faster than for the muscle. These differences reflect the much greater concentrations found in the liver compared to the muscle and further support the idea that uptake occurs first into the liver with some later redistribution to the muscle. Despite the much lower concentration in the muscle, the total amount of MC found in the muscle is often greater than that found in the liver because of the relative size of the two organs.

Higher concentrations of MC in the liver compared to the muscle tissue has been observed in many other studies as well, regardless of dosing mechanism or species [29,38,45,47,50]. Selective uptake of microcystins into hepatocytes occurs via active transport, resulting in organ specificity of accumulation [51]. Reasons for these differences in liver and muscle MC concentrations are demonstrated by the kinetics model developed in this study. The first order model used shows the rapid uptake and elimination of MC from the liver and much slower rates in the muscle. This is consistent with overall higher MC concentrations measured in the liver as well as the maximum liver concentrations proceeding maximum muscle concentrations in time. This leads to the hypothesis that the initial uptake of MC is into the liver and then partially redistributed into the muscle, prolonging the exposure period for the muscle. 

The kinetics of MC uptake and loss were similar regardless of dose, potentially driven by the ability of MC to cross membranes [22]. Without an increase in MC uptake rate with increased dose, total concentrations of MC in fish with a larger tissue mass would be overall lower than in smaller fish. Another toxicokinetic model, which was developed for accumulation of microcystins in the nile tilapia *Oreochromis niloticus*, showed that for lower doses of microcystins, there may be a greater dose dependent effect [45]. In this model, at doses of microcystins below 0.15 µg·MC·fish^−1^·day^−1^, accumulation in the liver was not accompanied by elimination, resulting in liver concentrations as high as 51% of the dose. At intermediate concentrations (0.15–2.5 µg·MC·fish^−1^·day^−1^), increasing available microcystins did not result in increased liver concentrations, due to an increased capacity for elimination. This model predicts that at concentrations greater than 2.5 µg·MC·fish^−1^·day^−1^, a saturation point is reached for nile tilapia and there is again an increase in liver microcystin concentrations with increasing dose [45]. Thus the lack of dose response for tissue concentrations in the current experiment may be an indication that doses of 5–20 µg MC fish^−1^ may be in the intermediate phase of accumulation for yellow perch of this size and that higher or lower concentrations of MC are necessary to see a dose response. 

### 2.4. Potential Impacts of Microcystins Human Health via Fish Consumption

The variability in exposure and the lack of data on the biological availability of the covalently bound microcystins in fish tissue makes it difficult to discern the potential risk of microcystins to human health through fish consumption [23,42]. There is circumstantial evidence of exposure to microcystins and toxicity to humans via consumption of contaminated fish [6] and the WHO recommended total daily intake (TDI) of 0.04 μg per kg bodyweight per day can be applied to concentrations of microcystins in fish to determine acceptable fish tissue concentrations. Populations that are in the greatest danger of health risks are those for which fish comprise a high percentage of the diet. Average consumption of sport fish by anglers and their families in the Great Lakes basin is estimated at 40 g fish·day^−1^, which is higher than the US national average fish consumption of 6.5 g day^−1^ [52,53,54]. Of greater concern are native tribal members for whom fish is much larger proportion of their diet, estimated to be an average of 190 g day^−1^ and as much as 328 g day^−1^ [55]. Using the WHO recommended TDI for a 70 kg adult and these average consumption rates, the maximum recommended fish muscle concentration would be 70 ng MC g fish^−1^ for the average Great Lakes angler, 14.7 ng MC g^−1^ for tribal members and 8.5 ng MC g^−1^ for very high fish consumers to prevent potential human illness. A recent draft document from EPA (NCEA-C-1765) used data from Heinze [56] to suggest a lower recommended limit for chronic exposure of 0.003 μg kg^−1^ per day, which would lower the maximum recommended fish levels of microcystins to 5.3, 1.1, and 0.6 ng MC g^−1^ for Great Lake anglers, tribal members and very high fish consumers, respectively. These recommended microcystin concentration would be even lower for children, elderly, or sensitive individuals. 

The uptake and elimination experiments described in this manuscript suggest that juvenile yellow perch exposed to environmentally relevant doses of microcystins have the capacity to eliminate the unbound fraction in the muscle tissue from a maximum of 18.7 ng g^−1^ ww down to 0.6 ng g^−1^ ww (expressed as wet weight to more accurately compare to the weight of a fish meal instead of dry weight as reported in the results) by 24 h after exposure. These data suggest that during periods of significant microcystin-producing blooms, consumers for whom fish is a substantial part of their diet could receive a dose that exceeds the WHO TDI. If the more conservative EPA guidelines were adopted, the recommended limits for chronic microcystins would be exceeded even by the average Great Lakes fish consumer during a toxic bloom. However, the evidence of rapid elimination presented here suggests that during non-bloom periods, muscle concentrations of unbound microcystins in yellow perch are likely lower than the recommended limit for chronic exposure. 

Poste *et al.* [40] also concluded that despite the higher concentration of microcystins measured in the muscle tissue of some Great Lakes fish causing individual fish to be of potential human health concern, the shorter length of the bloom season, time for elimination and amount of fish consumed made it likely that most consumers of Great Lakes fish were not receiving chronic exposure to microcystins through fish in excess of the WHO TDI. In contrast, an important finding in a study of concentrations of microcystins in fish from lakes in Uganda is that in communities in tropical locations with persistent year-round cyanobacterial blooms, which rely on local fish for subsistence and frequently consume the entire fish, may be at risk for health risks through chronic microcystin exposure [40]. 

## 3. Experimental Section

Juvenile yellow perch (*Perca flavescens*) were used for two uptake and elimination experiments, run in June and July 2008. Prior to each experiment, fish 9–14 cm in length were obtained at the Stoney Creek Fish Hatchery (Grant, MI) and kept in the dark with Stress Coat^®^, a synthetic slime coating which helps protect the fish, during the two hour transport to the laboratory. Fish were held at 20 °C for 24–28 h before the start of the experiment and were fed once. 

Microcystin LR was obtained from Sigma Scientific and stored at −20 °C. The day of the exposure, microcystin-LR (MC-LR) was dissolved in 100% methanol (MeOH) to the appropriate concentration, added to a food pellet (Zeigler salmon crumbles, Zeigler Bros. Inc. Gardners, PA) by pipette, and allowed to dry. Each fish was fed one pellet orally, thus delivering a known dose of MC-LR. Control fish were fed a pellet treated with just MeOH and no MC-LR. Fish were observed for 30 min to make sure the pellet was not regurgitated. The dose given was 20 µg MC-LR in the June experiment and 5 µg MC-LR in the July experiment. Dose concentrations were confirmed by analysis of extra food pellets.

Water used in all experiment was collected from the Huron River upstream from Dexter, MI at the Hudson Mills Metropark. Hardness (165–250 mg L^−1^ CaCO_3_), alkalinity (17–250 mg L^−1^ CaCO_3_) and pH (8.1–8.3) were measured before the experiments were performed. Four replicate fish were used for each time point and each fish was kept in an individual tank containing 4 L of filtered (0.2 µm filter) river water. In order to determine the time points for these experiments, a preliminary experiment was run in which the fish were dosed with 5 µg MC-LR, and measurements of microcystin concentrations in the liver and muscle were taken out to 240 h (at 2, 4, 8, 12, 24, 48, 72, 120, 168 and 240 h). Based on the results of this preliminary experiment, the period from 0–24 h was selected for more detailed analysis. At each time point (0, 2, 4, 6, 8, 10, 12, 15, 17, 19, 21, and 24 h), four fish were sacrificed, length and wet weight of each fish was recorded, and then liver and muscle tissues were removed for microcystin analysis. The entire fillet from each side of the fish was used for the muscle sample and the two fillets per fish were analyzed separately. The liver was run as a single sample. To control for factors outside dosing with microcystin, there were two sets (4 each) of control fish that were fed pellets without MC-LR and then processed in the same manner as the dosed fish; one set was taken for analysis at 0 h and the other at 24 h.

After the fish was removed from its tank at a given time point, the 4 L of tank water was transferred to a 4 L bottle and hooked up to a vacuum manifold. To concentrate the dissolved fraction of MC that had been excreted by the fish, the water was drop wise siphoned through a 3 cc Oasis HLB type SPE column (Waters, Milford, MA) that had been conditioned with 1 mL of 100% MeOH and equilibrated with 1 mL nano-pure water. If present, feces were not separated from tank water. The SPE column was then washed with 1 mL of 5% MeOH in water and samples were eluted with 100% MeOH, dried under vacuum at room temperature and stored at −20 °C. 

Fish tissues (liver, muscle) were freeze dried, weighed, and then extracted in 20 mL of 75% MeOH with mastication, using a variation of the method described in Wilson *et al.* [28]. Samples were centrifuged (9000 rpm, 5 min) and the supernatant retained in a clean glass vial. The tissue was extracted a second time in 30 mL of 75% MeOH plus 75 μL of acetic acid overnight. Again the sample was centrifuged and the supernatant combined with the supernatant from the first extraction, then dried under vacuum at room temperature. When dry, the extracted sample was placed under nitrogen, capped and stored at −20 °C until analyzed. For microcystin analysis, tissue extracts were rehydrated with nano-pure water and sonicated in an ice bath for 30 min to assist in dissolving dried sample. Microcystin analysis was by ELISA, according to manufacturer’s instructions (Envirologix, Portland, ME). The Envirologix assay uses a polyclonal antibody that detects multiple microcystin variants, with varying degrees of cross-reactivity. While the ELISA data is expected to be predominantly MC-LR since this is what was dosed into the fish, the ELISA data is expressed as concentration of microcystins (MC) to reflect the potential for other variants present. Microcystin-LR loss in the extraction process was quantified by measuring recovery of a known concentration of MC-LR spiked into non-dosed tissues from fish not included in the experiments. The average for the four fish at each time point was divided by the known initial dose delivered through the food pellet to get a percentage of the initial dose accounted for at each time point, which would not include microcystins present in the bile, gut, or other organs or covalently bound to the liver and muscle tissue. 

The kinetics of the uptake of this single dose of MC-LR and subsequent accumulation in muscle and liver over time as measured by immunoassay was modeled and used all available data including initial background concentrations. In order to determine the appropriate modeling approach to use, a preliminary analysis of the data was conducted. From a kinetic stand point, the exposure is a pulse and assumed to decline in a first order manner over time. A plot of the tissue MC concentration over time showed an increase to a peak and then a decline that appeared to be first order. Thus, there are three variables that affected the concentration of MC in the tissues: the exposure concentration which is assumed to decline in a first order manner, the accumulation and distribution of the MC to the tissues, and the biotransformation and elimination of MC from the tissue. Since an immunoassay was used for the measurements, all the material measured as MC was assumed to be parent compound but may include a metabolite(s) that is immunologically active. The approach to modeling the kinetics is limited because there are three variables and the tissue concentrations depend on each of those variables, so a direct fit was not thought to be the most practical approach to obtaining kinetic estimates. Since there is an apparent first order decline in tissue concentration beginning at 10 to 12 h after dosing, the declining tissue concentration was fit to a first order loss model (Equation 1) making the assumption that uptake was insignificant after that point in time. 


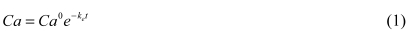


Here *Ca* is the concentration in the tissue (ng g^−1^ dw), *Ca*^0^ is the initial concentration in the tissue at the beginning of the loss process (ng g^−1^ dw), *t* is time (h) and *k_e_* is the elimination rate constant (h^−1^). 

The apparent elimination constant determined in this manner was then set as a fixed value and the tissue data modeled using a declining source term, as shown in Equation (2):


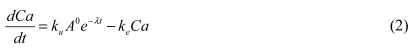


which integrates to Equation (3) and this equation is used to fit the data.


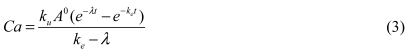


Here *Ca* is the concentration in the tissue (ng g^−1^ dw), *k_u_* is the uptake rate constant (g^−1^·h^−1^), *A*^0^ is the amount of compound dosed to the fish (ng), *k_e_* is the elimination rate constant (h^−1^) determined from equation 1, λ is the loss rate for the dose (h^−1^) and *t* is time (h). For both equations, the data are fit using Scientist^®^ (St. Louis, MO, USA).

The half life (*t*_0.5_) of the MC in each tissue was calculated based on the elimination rate constant *k_e_* (h^−1^) as shown in Equation (4):


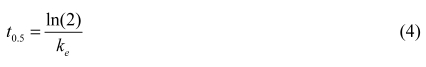


as well as the time in which 95% of the MC is eliminated from the fish tissue, as shown in Equation (5):


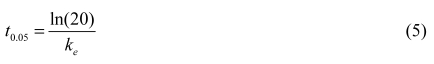


## 4. Conclusions

The goal of this study was to determine the rates and time scales of MC-LR uptake and elimination in yellow perch to help assess the potential for human exposure to this cyanotoxin through fish consumption. The relatively rapid uptake and elimination of the unbound fraction of MC in the liver and muscle of juvenile yellow perch within 24 h of oral exposure indicates that fish consumption may not be a major route of human exposure to microcystins in the Great Lakes. This efficient elimination of MC may also help explain the tolerance of fish to toxic *Microcystis* blooms, though does not eliminate the possibility of chronic impacts to fish health or the risk of bound microcystins in fish tissue to human health. 
